# The unusual isolation of carnobacteria in eyes of healthy salmonids in high-mountain lakes

**DOI:** 10.1038/s41598-021-82133-3

**Published:** 2021-01-27

**Authors:** Paolo Pastorino, Silvia Colussi, Elisabetta Pizzul, Katia Varello, Vasco Menconi, Davide Mugetti, Mattia Tomasoni, Giuseppe Esposito, Marco Bertoli, Elena Bozzetta, Alessandro Dondo, Pier Luigi Acutis, Marino Prearo

**Affiliations:** 1 The Veterinary Medical Research Institute for Piemonte, Liguria and Valle d’Aosta, via Bologna 148, 10154 Turin, Italy; 2 Department of Life Sciences, University of Trieste, via L. Giorgieri 10, 34127 Trieste, Italy; 3 Department of Veterinary Medicine, University of Sassari, via Vienna 2, 07100 Sassari, Italy

**Keywords:** Environmental microbiology, Freshwater ecology, Molecular biology

## Abstract

Carnobacteria are common bacteria in cold and temperate environments; they are also reported during fish mortality events. In a previous study, carnobacteria were isolated from the eyes of healthy wild salmonids from a high-mountain lake. To better understand these findings, salmonids were captured from three high-mountain lakes (Lower and Upper Balma Lake, Rouen Lake; northwest Italy) during August 2019 and subjected to bacteriological and histological examination. Although all were healthy, 8.7% (Lower Balma Lake), 24% (Upper Balma Lake), and 32.6% (Rouen Lake) were positive for carnobacteria colonization of the eyes. A Trojan-horse effect was hypothesized to explain carnobacteria isolation in the eye. This immune-escaping macrophage-mediated mechanism has been identified in other Gram-positive bacteria. Biochemical, molecular, and phylogenetic analysis were carried out on isolated bacteria (*Carnobacterium maltaromaticum* and *C. divergens*). Based on previous references for carnobacteria isolated from fish, *C. maltaromaticum* strains were tested for the *pisA* precursor gene of the bacteriocin piscicolin 126. *Carnobacterium maltaromaticum* strains were found to display genotypic heterogeneity and a low percentage of *pisA* positive amplification. Features of geomorphology, geographic isolation, and microbiota common to the three lakes are thought to be possibly related to our findings. Moreover, terrestrial insects collected from the lake shoreline and the stomach contents were screened for the presence of carnobacteria. The salmonids in these high-mountain environments feed mainly on terrestrial insects, which are considered possible vectors for carnobacteria that might catabolize the exoskeleton chitin. All insects tested negative for carnobacteria, but as a small number of samples were analyzed, their role as possible vectors of infection cannot be excluded. Further studies are needed to corroborate our research hypothesis.

## Introduction

Lactobacilli are common bacteria in cold and temperate environments and though normal components of the gastrointestinal microbiota of fish^1^ they are also associated with morbidity and mortality^2,3^. Based on phenotypic and molecular characterization, some lactobacilli have been renamed and categorized in the genus *Carnobacterium*, currently comprising 12 species^4^. Carnobacteria have been widely studied for their involvement as protective cultures that inhibit the growth of pathogens in chilled seafood and meat products^5^ and as potential fish probiotics^6^.

*C. maltaromaticum* (formerly *piscicola*) and *C. divergens* have been associated with infections in fish^7,8^. *C. maltaromaticum* was recognized as pathogen for Australian salmonids^9^, carp^10^, rainbow trout^8,11^, striped bass, and channel catfish^11,12^. Signs of infection vary from septicemia to healthy appearance, with virulence appearing under stress conditions. *Carnobacterium divergens* has been reported in Atlantic cod, Atlantic salmon^13^, trout, and freshwater fish^14–16^.

Molecular genetics and mechanisms of action of bacteriocins produced by carnobacteria useful for industrial purposes have been extensively studied^17^. Most of these antimicrobial peptides belong to class IIa, which also include piscicolin 126, reported for the *C. maltaromaticum* JG126 strain^18^. Class II bacteriocins are synthetized as inactive pre-peptides that are activated after cleavage of the N-terminal peptide leader. Piscicolin 126 is encoded by an operon located on the bacterial chromosome, including 7 genes: one encoding the bacteriocin aminoacidic sequence and the others encoding accessory functions such as immunity protein, ATP-binding cassette transport system (ABC), and its membrane-bound accessory protein^5^.

A previous study assessing the biological and health conditions of salmonids from a high-mountain lake reported the unusual presence of two species of carnobacteria (*C. maltaromaticum* and *C. divergens*) in the eyes of apparently healthy wild brook trout (*Salvelinus fontinalis*)^19^. To better understand these findings, during August 2019 fish sampling campaigns were performed in three high-mountain lakes (Upper Balma Lake, Lower Balma Lake, Rouen Lake) located in the Cottian Alps (northwest Italy). The main aim of this study was to isolate and identify carnobacteria from fish by means of culture, biochemical, and more reliable biomolecular techniques^20^, providing hypotheses about their presence only from the eye of apparently healthy fish.

Based on references about carnobacteria isolated from fish inhabiting lake ecosystems^21,22^, *C. maltaromaticum* strains were tested for the *pisA* precursor gene of piscicolin 126. Specific PCR primers were designed to study the *pisI* gene*,* located on the same operon, and encoding the immunity protein. Phylogenetic analysis of all strains was carried out.

Terrestrial insects collected around the lake shoreline and the stomach contents were screened for carnobacteria; the salmonids in these high-mountain environments feed mainly on terrestrial insects^19^, which are reported as a possible vector for carnobacteria that may catabolize the exoskeleton chitin^23,24^.

The high-altitude lakes included in this study are among the most remote aquatic environments in Europe. They are characterized by few but well-adapted native organisms (i.e., phytoplankton, zooplankton, macroinvertebrates)^25^. Their small size and high turnover of surface waters render these ecosystems extremely receptive and vulnerable to anthropogenic impacts, such as the introduction of alien fish^25^. Originally fishless, fish were released into the lakes several years ago for recreational fishing. Since then, they have survived and proliferated in extreme conditions (i.e., cold temperatures, UV exposure, ice cover for several months of the year) and with a bacterial community composition proper to these environments^26^.

## Results

**Figure 1:**
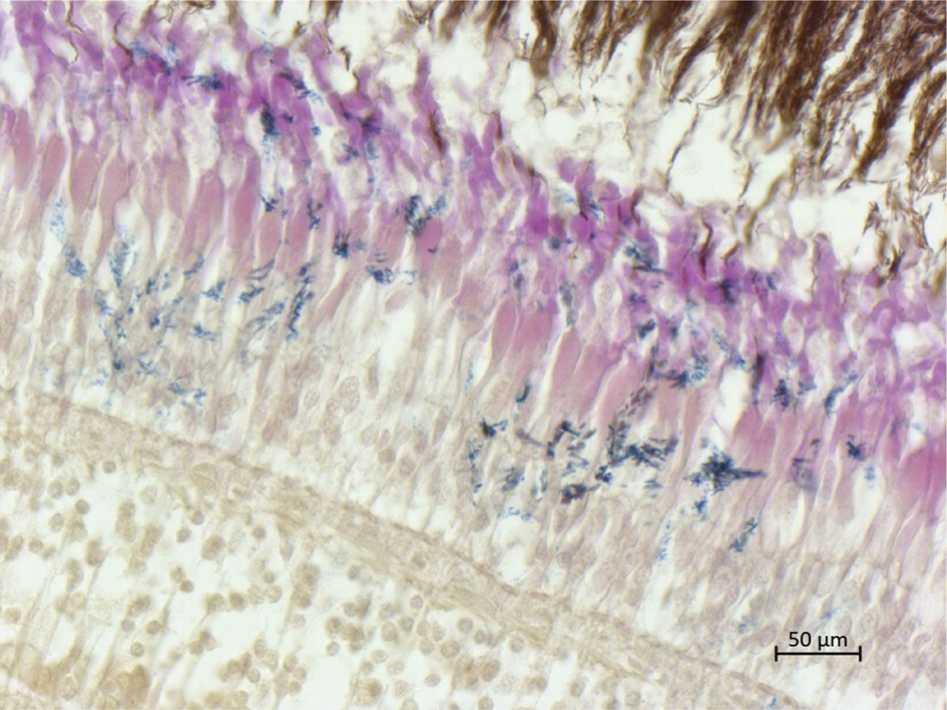
Eye: Gram-positive bacteria in the retina outer layers (Brown and Brenn gram stain).

Table 1 presents the biometric features of the fish and the results of bacteriological examination. All the fish appeared healthy, as confirmed by the absence of internal and external lesions at necropsy and histological analysis. Histological examination with Brown and Brenn gram staining revealed Gram-positive bacteria in all the eyes of the fish testing positive for carnobacteria with specific localization in cornea, retina and choroid rete (Fig. 1). This was confirmed by bacteriological examination: 24% and 8.7% of the brook trout from Upper and Lower Balma Lake, respectively, and 32.6% of the brown trout from Rouen Lake tested positive for carnobacteria colonization of the eyes. The other tissues (kidney and brain) resulted negative at bacteriological examination in all fish (Table 1).

**Table 1:** Total length (TL; cm), total weight (TW; g), and results of bacteriological examination (MALDI-TOF MS; ID: 99.9%) of kidney, brain, and eye of fish from the three lakes. Plus-minus values are the mean ± standard deviation. BT = bacteriological test; P (%) = prevalence of bacteria colonization in percentage; CM = *Carnobacterium maltaromaticum*; CD = *Carnobacterium divergens*.

Site	TL (cm)	TW (g)	Kidney		Brain		Eye	
			BT	P (%)	BT	P (%)	BT	P (%)
Lower Balma Lake	19.08 ± 3.57	78.80 ± 33.22	–	–	–	–	CM	8.7
Upper Balma Lake	21.68 ± 2.38	141.08 ± 39.76	–	–	–	–	CM; CD	24
Rouen Lake	23.88 ± 1.69	148.72 ± 31.99	–	–	–	–	CM	32.6

MALDI-TOF MS identified 4, 6, and 15 strains as *C. maltaromaticum* (ID 99.9%; n = 25) from fish from Lower Balma Lake, Upper Balma Lake, and Rouen Lake, respectively. *Carnobacterium divergens* strains (ID 99.9%; n = 9) were isolated only from Upper Balma Lake (Table 1). Morphological and biochemical analysis showed that all 34 isolates were Gram-positive bacilli, non-motile, negative for catalase and cytochrome oxidase, hydrolyzed esculin, produced arginine dihydrolase and 2,3-butanediol from glucose (Voges–Proskauer test), and did not reduce nitrate. None of the isolated strains produced indole, lysine decarboxylase, ornithine decarboxylase, urease nor were they able to utilize citrate. Generally, the isolates produced acid from glucose, mannitol, sucrose, and trehalose. No acid was produced from arabinose, galactose, inositol, maltose, raffinose, rhamnose, and sorbitol (Table 2); no biochemical differences were found between *C. maltaromaticum* and *C. divergens* isolates (Table 2).

**Table 2:** Biochemical characteristics of *Carnobacterium maltaromaticum* (CM) and *C. divergens* (CD) isolated from the three high-mountain lakes, as well as those of other carnobacteria strains and closely related carnobacteria (*Carnobacterium* spp.; CSP) recovered from fish species. +, positive reaction; −, negative reaction; (+), weak and/or delayed positive reaction; [+], 76–89% positive; [−], 11–25% positive; d, 11–89% positive; NR, not reported.

Biochemical test	Lower Balma Lake	Upper Balma Lake		Rouen Lake	Loch et al.^22^	Loch et al.^21^	Mora et al.^46^	Hiu et al.^47^	Baya et al.^12^	Starliper et al.^8^	Toranzo et al.^11^	Ringø et al.^48^	ATCC 27865
Bacteria	CM	CM	CD	CM	CM	CM	CM	CM	CSP	CM	CSP	CD	CM
Gram stain	+	+	+	+	+	+	+	+	+	+	+	+	+
Production of H_2_S	−	−	−	−	−	−	−	−	−	−	−	−	−
Citrate	−	−	−	−		−	NR	NR	−	−	−	NR	−
Production of indole	−	−	−	−	−	−	NR	NR	−	NR	−	NR	−
Urease	−	−	−	−	NR	NR	NR	NR	NR	NR	NR	−	NR
Esculin hydrolysis	+	+	+	+	+	+	+	+	+	+	+	NR	+
Voges–Proskauer	+	+	+	+	+	d	[+]	[+]	+	−	+	NR	(+)
Nitrate reduction	−	−	−	d	−	−	−	−	−	−	−	NR	−
Lysine decarboxylase	−	−	−	−	−	−	NR	NR	−	−	−	NR	−
Ornithine decarboxylase	−	−		−	−	−	NR	NR	−	−	−	NR	−
Arginine dihydrolase	+	+	+	+	+	+	+	+	+	+	+	NR	−
Catalase	−	−	−	−	−	−	−	−	−	−	−	−	−
Oxidase	−	−	−	−	−	−	−	−	−	−	−	−	−
ONPG	d	d	−	+	+	+	NR	d	+	d	+	NR	+
**Acid production from**													
Arabinose	−	−	−	−	−	−	NR	−	−	−	−	NR	−
Galactose	−	−	−	−	+	+	+	d	+	d	NR	NR	+
Glucose	+	+	+	+	+	+	+	+	+	+	NR	+	+
Inositol	−	[−]	−	−	NR	−	NR	−	NR	[−]	−	NR	−
Inulin	d	d	d	−	+	d	NR	−	+	NR	+	NR	+
Lactose	d	d	d	+	+	+	+	d	+	d	−	NR	+
Maltose	−	−	−	−	+	+	+	+	+	+	NR	NR	+
Mannitol	+	+	+	+	+	d	+	[+]	+	d	+	NR	+
Mannose	−	d	d	−	+	+	+	+	+	+	NR	NR	+
Melibiose	−	−	−	d	NR	+	NR	d	+	d	NR	NR	+
Raffinose	−	−	−	−	NR	−	NR	d	+	d	NR	NR	−
Rhamnose	−	−	−	−	−	−	NR	−	−	−	NR	NR	−
Sorbitol	−	−	−	+	+	−	NR	−	+	d	(+)	NR	−
Sucrose	+	+	+	+	+	+	+	+	+	+	+	NR	+
Trehalose	+	+	+	+	+		+	+	+	+	NR	NR	+

PCR amplicons of the intergenic spacer region (ISR) of gene 16S and 23S rRNA from the 34 carnobacteria isolates and *pisA* precursor gene of three *C. maltaromaticum* out of the 25 isolates were sequenced. *Carnobacterium maltaromaticum* isolates from the fish from Upper Balma (n = 6) and Rouen Lake (n = 10) were highly similar to *C. maltaromaticum* GenBank reference sequence AF374295 (BLASTn similarity is reported in Table 3). All the strains were identical to each other; for this reason, the consensus sequence (called Cm1, GenBank Accession number MW447308) was entered into phylogenetic analysis. Three *C. maltaromaticum* isolates from the fish from Lower Balma Lake were identical to each other but different from the *C. maltaromaticum* isolated from the fish from Upper Balma Lake and were more similar to *C. maltaromaticum* reference sequence AF374297 (Table 3); the consensus sequence (called Cm2, GenBank Accession number MW447302) was entered into phylogenetic analysis. One isolate from the fish from Lower Balma Lake (Cm3, GenBank Accession number MW447306) differed from the three other isolates by a single mismatch at position 606 (a > c) referred to reference sequence AF374297 (Table 3). Five *C. maltaromaticum* isolates from the brook trout from Rouen Lake were identical to each other (100% identity with *C. maltaromaticum* strain LMA28 according to BLASTn) but differed from AF374295 by 11 mismatches (Table 3); the consensus sequence (called Cm4, GenBank Accession number MW438292) was entered into phylogenetic analysis. Seven out of nine *C. divergens* isolates from the fish from Balma Lake were highly similar to *C. divergens* GenBank reference sequence AF374294 (BLASTn similarity 100%, expectation value 5e−160). All the strains were identical to each other; for this reason, the consensus sequence (called Cd1, GenBank Accession number MW447309) was entered into phylogenetic analysis. Two *C. divergens* isolates were identical to each other but different from the other *C. divergens* isolates and from the reference sequence by a single mismatch at position 447 (t > a) referred to reference sequence AF374294 (BLASTn similarity 99.68%, expectation value 3e−158). The consensus sequence (called Cd2, GenBank Accession number MW447307) was entered into phylogenetic analysis.

**Table 3:** BLASTn identity values of the consensus obtained for *C. maltaromaticum*.

Strain	Identity percentage with GenBank AF374295	Identity percentage with GenBank AF374297
Cm1	99.81	93.72
Cm2	94.49	100.00
Cm3	94.45	99.78
Cm4	97.39	95.98

Only three out of 25 *C. maltaromaticum* (12%) (two isolates from Upper Balma Lake and one from Rouen Lake) were positive for the *pisA* precursor gene. The sequences were identical to each other and to *pisA* reference sequence AF275938 (BLASTn similarity 99.63%, expectation value 2e−134), while less similarity was found for the *pisA* precursor gene of *C. maltaromaticum*-like bacteria from lake whitefish (EU643471) reported by Loch et al.^21^ (BLASTn similarity 98.51%, expectation value 1e−130). A *pisI* 728 bp amplicon was obtained only for one out of three *C. maltaromaticum pisA-*positive isolates from the fish from Upper Balma Lake.

**Figure 2:**
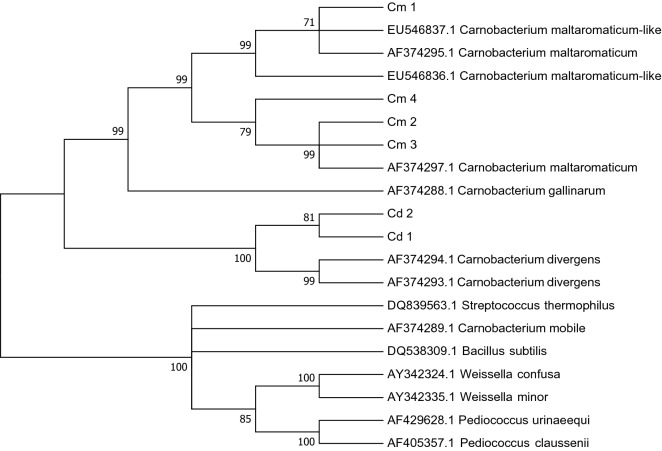
Evolutionary relationships of taxa based on ISR. The evolutionary history was inferred using the neighbor-joining method. Shown is the optimal tree with the sum of branch length of 1.24457936. The percentage of replicate trees in which the associated taxa clustered together in the bootstrap test (1000 replicates) are shown next to the branches; only bootstrap values > 70 are displayed. The evolutionary distances were computed using the maximum composite likelihood method and are expressed as units of the number of base substitutions per site. Analysis involved 20 nucleotide sequences. Codon positions were 1st + 2nd + 3rd + Noncoding. All positions containing gaps and missing data were eliminated. There was a total of 200 positions in the final dataset. Evolutionary analyses were conducted in MEGA7.

Phylogenetic analysis (Fig. 2) showed intraspecies genotypic variability within the *C. maltaromaticum* clade. Two *C. maltaromaticum* clusters were present: one comprising Cm1, *C. maltaromaticum* reference sequence AF374295, and the *C. maltaromaticum-like* reference sequences EU546837 and EU546836, and the other comprising Cm2, Cm3, Cm4, and *C. maltaromaticum* reference sequence AF374297. Of interest was the node (bootstrap value 79) that separated two *C. maltaromaticum* subgroups from Cm2 and Cm3 together with reference sequence AF374297 and Cm4 standing alone. The prevalence of distribution of Cm1, Cm2, Cm3, and Cm4 in the three lakes was statistically significant (Fisher’s exact test; *p* < 0.001).

*Carnobacterium gallinarum* belong to the same clade as *C. maltaromaticum*, as reported by Loch et al.^21^, while *C mobile* was completely separated. *C. divergens* isolates created a well-separated different clade with intraspecies genotypic variability, as shown by the node (bootstrap value 81) that separates Cd1 from Cd2.

Terrestrial insects of the orders Imenoptera (families Vespidae 75% and Formicidae 5%) and Coleoptera (Carabidae family 20%) were found in the stomach contents (n = 128) of the fish from the three lakes; 5 specimens of *Carabus cenisius fenestrellanus* (Coleoptera; Carabidae) were collected from the pitfalls placed along the shoreline of Lower Balma Lake.

No carnobacteria were identified based on insect DNA extraction; one insect in the stomach content was infected with *Spiroplasma monobiae* (BLASTn similarity 98.82%, expectation value 0) described as a vespid strain^27^. Bacteria of the genus *Clostridium*, which is often reported as commensal bacteria in insects^28^, were detected in an insect from the stomach content.

## Discussion

Carnobacteria were frequently found in the wild brook trout and the brown trout from three high-mountain lakes. An unusual finding was that *C. maltaromaticum* and *C. divergens* were isolated in apparently healthy fish and limited only to the eye. Previous studies have described systemic infections, with frank symptoms in feral lake fish populations^21,22^. The carnobacteria from the brook trout showed the phenotypic and biochemical characteristics typical of this species^21,22^*.* The *C. maltaromaticum* strains displayed genotypic heterogeneity possibly related to the geomorphology and the ecosystems of the three lakes, as suggested by the statistically significant distribution of Cm1, Cm2, Cm3, and Cm4.

Different bacteriocins have been described for carnobacteria^17^. Though the molecular genetics and mode of action are well known for strains exploitable for industrial application, the selective forces driving their evolution are not well understood^17^. In this study we screened *Carnobacterium maltaromaticum* strains only for the presence of *pisA* so that we could compare our data with those reported by Loch et al.^21^. A low percentage of *C. maltaromaticum* were positive for the *pisA* precursor gene and the sequences had a high identity percentage with the GenBank reference sequence AF275938, related to the JG126 strain; whereas the *pisA* sequences from the lake whitefish described by Loch et al.^21^ differed by 4 single nucleotide polymorphisms.

The evolution of bacteriocin production is often influenced by mutual interactions among bacteria^17^; and it is often related to the metabolic cost for their production^17^. The geomorphology and geographic isolation of the three lakes could account for the differences in the composition of the bacteria community, with a possible impact on bacterial interaction and metabolic needs. For example, piscicolin has been shown to act as a bactericidal for other Gram-positive bacteria such as *Listeria monocytogenes*. A study conducted in Austria reported that the most dominant species of the genus *Listeria* in soil and water samples were: *Listeria seeligeri, L. innocua, and L. ivanovii,* while *L. monocytogenes* was isolated in only 6% of soil samples from a mountainous region^29^. The piscicolin 126 operon might have undergone destruction over time by insertion, rearrangement or replacement ^30^. The absence of amplification products for the immunity gene *pisI,* also in positive *pisA* strains, and the consequent absence of immunity to piscicolin 126 also in producer strains, could be considered an indicator of this process.

The finding of carnobacteria isolated only from the eye might be interpreted in the context of immune-escape mechanism adoption, such as a macrophage-mediated Trojan-horse effect, which has been described for other gram-positive bacteria^31^. This effect in fish has been observed for *Streptococcus iniae* that enter and multiply within macrophages where the pathogen gains an efficient mechanism for translocation into the central nervous system^32^. The eye provides a protected environment for bacteria that can then infect wild populations under favorable conditions, such as those of the postspawning period, which previous studies described as a stressful period associated with greater predisposition to disease^7,8,33^. Vitreal macrophages, called hyalocytes, are primarily located between the inner membrane of the retina and the vitreous membrane, which encapsulates the condensed vitreal collagen^34^. For the present study, sampling was done in the pre-spawning period when stress is low. Sampling during postspawning would have been unfeasible because the three lakes are covered by ice for several months of the year (generally from late October to June)^35^.

*Carnobacterium maltaromaticum* is characterized by chitinolytic activity, i.e., the ability to catabolize chitin, a molecule in the insect exoskeleton; and insects have indeed been reported as hosts for this species^23,24^. Since the terrestrial insects provide the main food source for fish in high-altitude lakes^19^, they were screened as a possible route of infection. The absence of carnobacteria in the terrestrial insects and those collected from the stomach content does not preclude them as a source of carnobacteria infection. Because of the small number of insects sampled, we will now attempt to increase the number of samplings for an extended range of species for the ISR amplification.

It was found that the temperate/polar aquatic and terrestrial environments are both natural habitats for carnobacteria, especially for *C. maltaromaticum* and *C. divergens*^1^. Indeed, these latter were recorded in polar and sea, cold and alkaline tufa columns, Japanese lakes and *Sphagnum* pond^1^. Despite this, the mechanisms by which carnobacteria occur and persist in the natural environment and their genetics are still unknown. Moreover, distribution, quantitative microbial ecology and genomics are important areas where knowledge on these bacteria is limited. Thus, further studies are necessary to verify carnobacteria occurrence in sediment and water samples from high-mountain lakes as well as in other fish tissues (i.e., gills).

Further research will be carried out also to test the hypothesis of the present study and intra-species diversity identification by means of rep-PCR, reported as a useful method for *C. divergens*^36^, virulence genes^37^, and other bacteriocins genes. Moreover, primary culture from fish eye will be done to detect the presence of carnobacteria inside the macrophages.

## Methods

### Study sites

**Figure 3:**
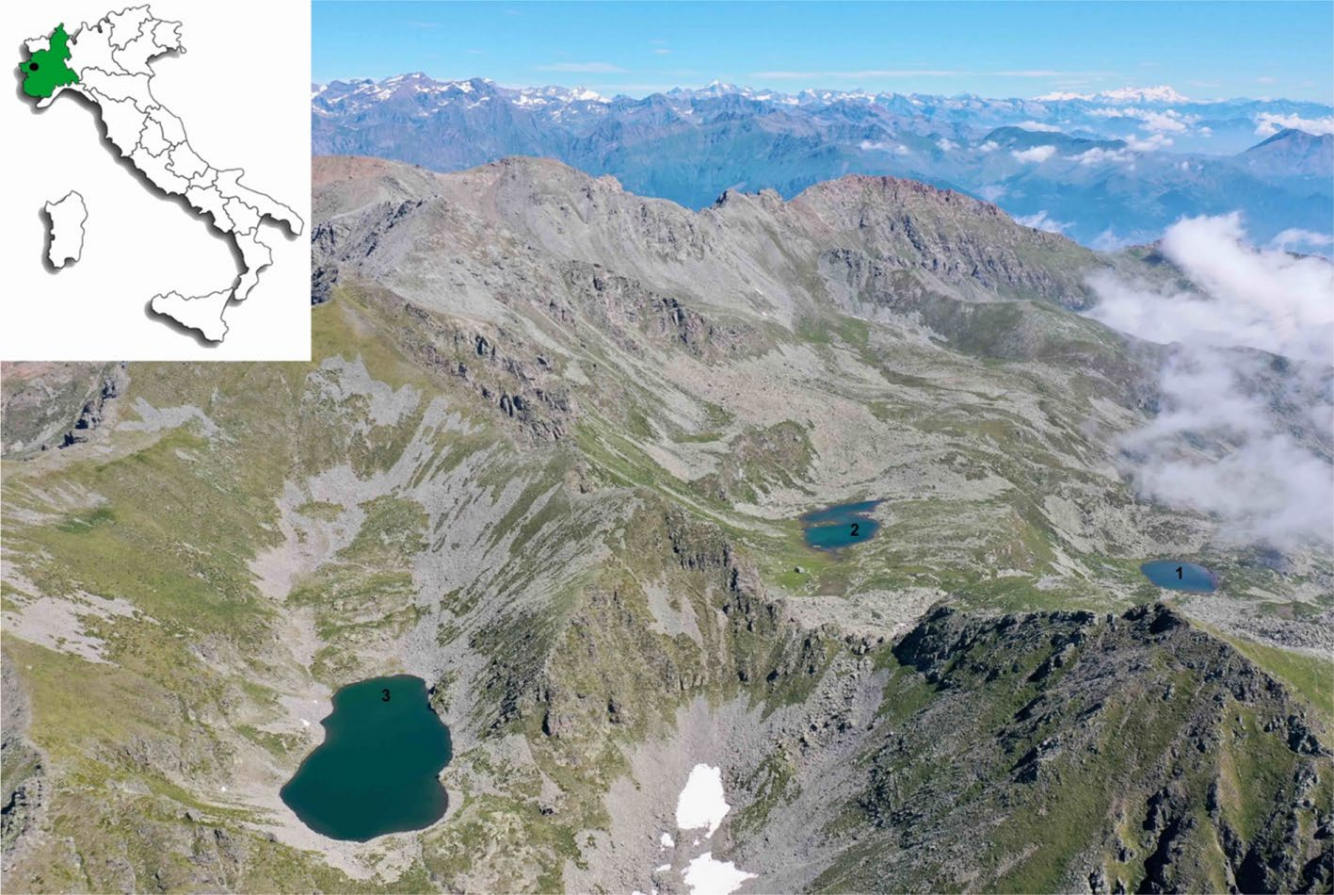
Location of fish sampling sites in the Cottian Alps (northwest Italy): (1) Lower Balma Lake, (2) Upper Balma Lake, and (3) Rouen Lake (drone photo: Paolo Pastorino).

The three lakes are located in the Site of Community Importance (SCI)/Special Areas of Conservation (SAC) IT1110006—Orsiera Rocciavrè (Fig. 3). Upper (2211 m above sea level-a.s.l.) and Lower (2100 m a.s.l.) Balma Lakes are located in the municipality of Coazze (Turin Province, northwest Italy); Rouen Lake (2391 m a.s.l.) is located in the municipality of Roure (Turin Province, northwest Italy). Table 4 presents the geographical coordinates, area, and depth of each lake. The lakes are situated above the tree line and covered by ice from late October to early June. The main anthropogenic pressures are grazing (cows in summer) and recreational fishing. Originally fishless, fish were introduced for recreational fishing in the past, probably in the 1980s^38^. The main core of the area is composed of ophiolite metamorphic bedrock. The physicochemical characteristics of the water indicate these lakes as oligotrophic environments^19^.

**Table 4:** Geographical coordinates, water free-surface altitude, surface, maximum depth of the lakes.

Lake	Coordinate	Water free-surface altitude (m a.s.l.)	Surface (ha)	Maximum depth (m)
Lower Balma Lake	45°02′14″N	2100	1.21	6.42
	07°10′52″E			
Upper Balma Lake	45°02′12.8″N	2211	0.58	4.27
	7°10′27.0″E			
Rouen Lake	45°01′47.2″N	2391	2.25	18.70
	7°10′00.0″E			

### Fish sampling and necropsy

During August 2019, specimens of brook trout (*S. fontinalis*) were captured from Upper Balma Lake (n = 25) and Lower Balma Lake (n = 57); 46 specimens of brown trout (*Salmo trutta*) were captured from Rouen Lake. The fish were captured using pelagic and benthic gillnets according to a standardized protocol proposed by Pastorino et al.^19^. The fish were euthanized with an overdose of tricaine methanesulfonate (MS-222; 250 mg L^−1^), measured for total weight (g) and total length (cm), and subjected to onsite external and internal health examination.

Permission for fish sampling and necropsy was obtained from the institutional review board “Funzione Specializzata Tutela Flora e Fauna” of Città Metropolitana di Torino (authorizations no. 176-19040/2017 and no. 309-8374/2019), as required by local laws. Experimental procedures were carried out according to the Guidelines of European Directive 2010/63/EU for the protection of animals used for scientific purposes, ARRIVE guidelines^39^ and the principle of the 3Rs was applied.

### Histological examination

Samples from the main organs (liver, kidney, spleen, brain, eyes) were collected and immediately fixed in 10% neutral buffered formalin after capture. The samples were then processed by standard paraffin wax techniques. All samples were cut in sections 4 ± 2 μm thick with a microtome and stained with hematoxylin and eosin to reveal histopathological features. Histochemical staining, Brown and Brenn (BB) gram stain for differential staining of gram-positive and gram-negative bacteria in tissue sections^40^ was performed on the eye tissues. Slides were inspected microscopically at increasing magnification (10 ×, 20 ×, 40 ×, 100 ×) on a Zeiss Axio Scope A1 microscope (Zeiss, Germany). The samples were considered positive for the presence of red or blue bacteria, identified as gram negative or gram positive, respectively.

### Insect sampling

To collect insects from the fish stomachs, the peritoneal cavities were carefully opened using individual sterile forceps/scissors and the stomach was removed. The stomach content was inserted in 15-mL conical Eppendorf tubes and stored on dry ice until analysis. The contents were then identified at the order or the family level by stereomicroscopy (Zeiss Stemis V8, Zeiss, Germany). Insects present in the stomach contents from five fish positive for carnobacteria were processed for molecular analysis.

Terrestrial insects were captured using four pitfall traps (6.5 cm in diameter and 10 cm in depth) placed along the shoreline of Lower Balma Lake. The traps were made from plastic bottles cut in two parts, the one inserted upside down into the other; the bottom was perforated and filled with 120 mL of white vinegar to keep the trapped insects alive until collection^41^. The insects were identified at the species or the family level and stored at − 20 °C until molecular analysis.

### Bacterial isolation and phenotypical and molecular characterization

Kidney, brain, and eyes from each fish were subjected to bacteriological examination immediately after capture and following standardized protocols^42^. Bacteriological examination of the eye entailed: (1) cleaning of the eye surface with absolute ethanol; (2) drilling of the eye surface with a glowing platinum inoculation loop, allowing the loop to cool before inserting it in the vitreous humor; (3) retraction of the loop while avoiding contamination; (4) streaking of the sampled tissue onto agar plates.

All primary cultures were incubated at 22 °C for up to 72 h on trypticase soy agar (TSA), Cresol red thallium acetate sucrose inulin agar (CTSI) (a selective and differential medium for *Carnobacterium* spp.)^21^ and Columbia blood agar (CBA); colonies were then sub-cultured onto TSA and incubated for 24 h at 22 °C for biochemical and phenotypic analysis, as reported elsewhere^21,22^. The isolates were maintained at − 80 °C in trypticase soy broth supplemented with 20% glycerol. Phenotypic bacteria identification was confirmed by matrix-assisted laser desorption ionization-time of flight mass spectrometry (MALDI-TOF MS) on a VITEK MS system (bioMérieux, France).

Twenty-five isolates identified as *C. maltaromaticum* and 9 identified as *C. divergens*, according to phenotypic and biochemical characterization, were subjected to molecular characterization. Pellets from colonies incubated at 22 °C for 24 h were used for DNA extraction.

DNA extraction was performed using the boiling and freeze-thawing protocol: briefly, a pellet was resuspended in R/DNAse-free water and heated for 15 min at 95 °C, followed by quick cooling on ice and pelleting down debris by centrifugation for 5 min at 7000*g* and using the supernatant. PCR amplification was performed using the primers for the intergenic spacer region (ISR) of the 16S and 23S of rRNA genes and the *pisA* precursor gene for the piscicolin 126 protein, as described by Pellè et al.^18^. Amplification of *pisI* was carried out in the *pisA* positive strains.

PCR of the ISR and the *pisA* precursor gene was carried out as described in Pellè et al.^18^ using a GeneAmp PCR System 9700 (Applied Biosystems, USA) with an initial denaturation step at 95 °C for 2 min, followed by 32 amplification cycles, with denaturation at 94 °C for 1 min, annealing at 56 °C for 1 min, elongation at 72 °C for 1 min, and a final extension step at 72 °C for 10 min. *Lactococcus garvieae* (DSM20684) and *C. maltaromaticum* (ATCC 27865) were used as negative and positive controls, respectively.

PCR of the ISR was carried out on the DNA samples extracted from the insects retrieved from the fish stomachs and the insects collected from the lakeshore of Lower Balma Lake, using a Qiamp Mini kit (Qiagen, the Netherlands) following the manufacturer’s instructions.

Amplicons were run on 2% Gelgreen (Biotium, USA) stained agarose gel and then visualized under UV exposure. A 50–2000 kb ladder (Amplisize Molecular Ruler, Bio-Rad, USA) was used as a molecular marker. PCR amplified a 600 bp band for the ISR and a 300 bp band for the *pisA* precursor gene, as described in Pellè et al.^18^. PCR amplified a 450 bp band for the ISR of *C. divergens*.

The amplicons were purified with an ExtractMe DNA Clean-up & Gel-out kit (Blirt, Poland) according to the manufacturer’s instructions. The purified PCR products were bidirectionally sequenced using Big Dye 3.1 (Applied Biosystems, USA) chemistry and the same primers used for PCR amplification. Cycle sequencing products were purified using Dye Ex 2.0 spin kit (Qiagen) and sequenced in an ABI3130xl Genetic analyzer (Applied Biosystems).

Additional primers were designed for *PisI* (immunity gene) amplification using on-line free software Primer3: *PisI* forward 5′-GACCTGATCATTTTGACGCATAT-3′ (annealing at 1134–1156 position of GenBank reference sequence AF275938) and *PisI* reverse 5′-AGCAGCAAATTTGACTACAGGT-3′ (annealing at 1840–1861 position of GenBank reference sequence AF275938).

The PCR reaction volume of 25 μl for each sample contained a final concentration of 240 nM for each primer, 0.2 mMol dNTPs mix (Fisher Molecular Biology, Italy), 1 U of platinum Taq (Invitrogen, USA), 1X PCR buffer without Mg^2+^, 1.5 mM MgCl_2_, and 20 ng of DNA template, with DNase-free water composing the remainder of the reaction mixture. DNA amplification was carried out on a GeneAmp PCR System 9700 (Applied Biosystems) with an initial denaturation step at 95 °C for 2 min, followed by 35 amplification cycles with denaturation at 94 °C for 1 min, annealing at 53 °C for 1 min, elongation at 72 °C for 1 min, and a final extension step at 72 °C for 10 min. Amplicons were run on 2% Gelgreen (Biotium) stained agarose gel and then visualized under UV exposure. A 50–2000 kb ladder (Amplisize Molecular Ruler, Bio-Rad) was used as a molecular marker.

### Molecular statistical analyses

Contig for each strain and consensus sequences obtained through multiple alignments were created using Lasergene SeqMan software (DNAStar). The generated contigs were analyzed using BLASTn software from the National Center for Biotechnology Information, (NCBI, USA) to detect homologous sequences. ISR consensus sequences for the type strains detected *C. maltaromaticum* (n = 25; 4 consensus sequences) and *C. divergens* (n = 9; 2 consensus sequences); the reference sequences (16S ribosomal RNA gene, partial sequence; 16S–23S ribosomal RNA intergenic spacer, complete sequence; 23S ribosomal RNA gene, partial sequence) from 14 bacterial taxa were aligned and neighbor-joining analysis was performed^43^ using Molecular Evolutionary Genetics Analysis software (MEGA; Ver. 7.0), with evolutionary distances ascertained via the maximum composite likelihood method^44^. A bootstrap test of 1000 replicates was performed.

Fisher’s exact test was applied to determine whether the prevalence of distribution of Cm1, Cm2, Cm3, and Cm4 in the three lakes was statistically significant. Descriptive statistics (mean ± standard deviation, SD) were used to describe the biometrical features of the fish. Results were considered statistically significant at a *p*-value < 0.05. Statistical analysis was performed using open-source data analysis software R (version 3.5.2.)^45^.

## Data Availability

All the sequences obtained for *Carnobacterium maltaromaticum* and *Carnobacterium divergens* have been submitted to GenBank database. The accession numbers are reported below: Cm1 (MW447308); Cm2 (MW447302); Cm3 (MW447306); Cm4 (MW438292); Cd1 (MW447309); Cd2 (MW447307). Other data that support the findings of this study are available upon request. Please contact the corresponding author (Paolo Pastorino).
